# Medical Costs and Productivity Losses of Cancer Survivors — United States, 2008–2011

**Published:** 2014-06-13

**Authors:** Donatus U. Ekwueme, K. Robin Yabroff, Gery P. Guy, Matthew P. Banegas, Janet S. de Moor, Chunyu Li, Xuesong Han, Zhiyuan Zheng, Anita Soni, Amy Davidoff, Ruth Rechis, Katherine S. Virgo

**Affiliations:** 1 Division of Cancer Prevention and Control, National Center for Chronic Disease Prevention and Health Promotion, CDC; 2 Health Economics Research on Cancer Workgroup; 3 National Cancer Institute, Bethesda, Maryland; 4 American Cancer Society, Atlanta, Georgia; 5 Agency for Healthcare Research and Quality, Rockville, Maryland; 6 Livestrong Foundation, Austin, Texas; 7 Emory University, Atlanta, Georgia

The number of persons in the United States with a history of cancer has increased from 3 million in 1971 to approximately 13.4 million in 2012, representing 4.6% of the population (1,2). Given the advances in early detection and treatment of cancer and the aging of the U.S. population, the number of cancer survivors is projected to increase by >30% during the next decade, to approximately 18 million (2,3). Cancer survivors face many challenges with medical care follow-up, managing the long-term and late effects of treatments (4), monitoring for recurrence, and an increased risk for additional cancers (4,5). These survivors also face economic challenges, including limitations in work and daily activities, obtaining health insurance coverage and accessing health care, and increasing medical care costs. To estimate annual medical costs and productivity losses among male and female cancer survivors and persons without a cancer history, CDC, along with other organizations, analyzed data from the 2008–2011 Medical Expenditure Panel Survey (MEPS), sponsored by the Agency for Healthcare Research and Quality. The results indicate that the economic burden of cancer survivorship is substantial among all survivors. For male cancer survivors, during 2008–2011, average annual medical costs and productivity losses resulting from health problems per person and adjusted to 2011 dollars were significantly higher among cancer survivors than among persons without a cancer history, by $4,187 and $1,459, respectively; for females, the estimated annual costs per person were $3,293 and $1,330 higher among cancer survivors than among persons without a cancer history, respectively. These findings suggest the need to develop and evaluate health and employment intervention programs aimed at improving outcomes for cancer survivors and their families.

For this report, data from the 2008–2011 MEPS (annual response rate = 53.5%–59.3%) and the 2011 MEPS Experiences with Cancer Survivorship Survey (6) (response rate = 90.0%) were analyzed. MEPS is an annual nationally representative survey of the U.S. civilian noninstitutionalized population that collects detailed information on demographic characteristics, health status, income, employment, and health-care expenditures. In 2011, cancer survivors (persons who self-report a cancer history) were asked to complete a supplemental questionnaire about the economic burden of cancer (6). Persons who only reported nonmelanoma skin cancer were not included in the cancer survivors group. All data were analyzed using statistical software, accounting for the complex survey design to obtain nationally representative estimates. Medical costs (total annual medical expenditures) and productivity loss among cancer survivors were estimated adjusting for age, sex, race/ethnicity, number of MEPS priority conditions, marital status, and education.

Total annual medical costs, stratified by sex, were estimated using annual medical expenditures among cancer survivors and persons without a cancer history. The estimated total annual medical costs were also examined by source of payment and service type. Lost productivity was estimated by assessing employment disability (being unable to work because of illness or injury), health-related missed work days, and days spent in bed because of ill-health, stratified by sex. Multivariable logistic regression was used to estimate the percentage of those unable to work because of illness or injury, adjusting for age, sex, race/ethnicity, number of MEPS priority conditions, and education. Negative binomial regression was used to estimate missed work days and days in bed. All medical costs and productivity losses were adjusted to 2011 dollars.

Indicators of productivity loss among cancer survivors were also examined using data from the 2011 MEPS Experiences with Cancer Survivorship Survey, stratified by sex. The percentage of cancer survivors employed at any time since their diagnosis, changes in work because of cancer, and limitations in physical and mental tasks at work, productivity at work, and daily activities outside of work were estimated using multivariable logistic regression, adjusting for age, sex, race/ethnicity, and number of MEPS priority conditions.

What is already known on this topic?

Cancer survivors have increased risk for additional cancers and often experience lasting and late effects of treatment. The economic burden of illness, including medical expenditures and productivity losses, can be significant because half of the estimated 13.4 million cancer survivors are of working age.

What is added by this report?

From 2008 to 2011, male cancer survivors incurred average annual medical expenditures of approximately $8,000 per person and per capita productivity loss of $3,700. For female, the estimates were $8,400 for annual medical expenditures and $4,000 for per capita productivity loss. Among men, these estimates were nearly two times higher and for women they were one-and-half times higher than among persons without a cancer history. Nearly 32% of survivors experienced limitations in their usual daily activities outside of work because of cancer and, among those employed, an estimated 42% had to make changes to their work hours and duties.

What are the implications for public health practice?

As the population of cancer survivors increases, the economic impact of cancer for patients, families, employers, the health-care system, and society overall is expected to grow. Given the increased health-care needs and medical costs of cancer survivors, continued access to health care and ways to reduce disruptions in work and daily activities are important when survivors complete their cancer treatment. Such efforts could reduce the economic burden caused by cancer and could help maximize employment opportunities and productivity among cancer survivors.

Cancer survivors were more likely to be female, non-Hispanic white, in fair/poor health and insured and to have multiple chronic conditions compared with persons without a cancer history (Table 1). During 2008–2011, male cancer survivors had mean annual medical expenditures of $8,091, compared with $3,904 among males without a cancer history (Table 2). Female survivors had mean annual medical expenditures of $8,412, compared with $5,119 among females without a cancer history. Among survivors, private health insurance was the largest source of payment ($3,003 and $3,899 for males and females, respectively), followed by Medicare. Ambulatory care medical services accounted for the largest share ($2,640 and $3,187) among survivors, followed by inpatient care ($1,722 and $1,843).

Among male cancer survivors, the per capita mean annual productivity loss was $3,719, compared with $2,260 among males without a cancer history (Table 2). For female survivors, the per capita mean annual productivity loss was $4,033, compared with $2,703 among those without a cancer history. Employment disability accounted for about 75% of productivity loss among male and female survivors.

Nearly one third of cancer survivors experienced limitations in their ability to perform usual daily activities outside of work, and 12% had impeded ability to perform mental tasks associated with usual daily activities (Table 3). Among cancer survivors who were employed at any time since diagnosis, cancer and its treatment interfered with physical tasks (25%) and mental tasks (14%) required by the job, with nearly 25% of cancer survivors feeling less productive at work. Although males were more likely than females to have been employed since their diagnosis (62% and 55%, respectively), among those employed, females were significantly more likely to make changes in work because of cancer than males (48% and 34%, respectively).

## Discussion

The results of this analysis indicate that overall, cancer survivors had total annual medical expenditures estimated at $4,187 more for males and $3,293 more for females, compared with those of persons without a cancer history. These estimates were adjusted for age, sex, race/ethnicity, number of MEPS priority conditions, marital status, and education. These findings add to the growing concerns about the costs of cancer treatment and their negative impact on cancer survivors and their families. For example, a recent study reported that persons diagnosed with cancer are at higher risk for bankruptcy than those without a cancer history (7). In 2012, the National Cancer Policy Forum of the Institute of Medicine (IOM) convened a workshop, “Delivering Affordable Cancer Care in the 21st Century” (8), to discuss the drivers of current and projected costs of cancer care and potential ways to curtail these costs and maintain high-quality care. In 2009, before the IOM workshop, the American Society of Clinical Oncology published a guidance statement on the cost of cancer care (9). Overall, these efforts underscore the growing recognition by medical professionals, including clinical oncologists, of the important role they play in reducing the cost of cancer care for cancer survivors. A 2013 IOM publication, *Delivering High-Quality Cancer Care: Charting a New Course for a System in Crisis*,* highlighted the importance of information about cancer costs and of quantifiying the economic issues encountered by cancer survivors and their families.

Many cancer survivors return to work and remain productive. However, for nearly a third of survivors, cancer and the lasting and late effects of treatment interfere with usual daily activities outside of work. Many of these survivors are in poor health. These survivors might be returning to work to maintain adequate health insurance coverage and to pay for cancer-related services not covered by insurance. For instance, approximately 10% of survivors aged <65 years in this analysis were uninsured (and therefore likely have incurred a larger personal financial burden) and might experience financial barriers to needed care than survivors who have some source of payment for medical services. The provisions of the Affordable Care Act are expected to help improve this situation by increasing access to health insurance for millions of persons living in the United States, including cancer survivors. Further, approximately 30% of survivors are disabled and not able to return to work or have decreased ability to work because of limitations in cognitive, mental, and physical functioning and psychological distress (10). These survivors are more likely to incur higher productivity losses than persons without a cancer history. These challenges, particularly those related to employment, might differ for men and women, as presented in this report.

The findings in this report are subject to at least five limitations. First, because of inadequate sample size, these analyses were not stratified by cancer site or by time since diagnosis. Second, other aspects of economic burden of illness were not included, such as the time spent receiving medical care, productivity losses for caregivers, and intangible costs associated with pain and suffering from cancer and its treatment. Therefore, the reported medical and productivity costs represent only a portion of the total economic burden of cancer to society, survivors, and their families. Third, this analysis relied on self-report of cancer diagnosis, which was not verified by medical records, and household-reported survey data, which are subject to measurement errors (e.g., underreporting). Fourth, because the 2008–2011 MEPS response rates were <60%, the findings might reflect, in part, nonresponse bias. Finally, because the MEPS priority conditions were based on a count of 10 conditions, some of the burden attributable to cancer could be attributed to unmeasured comorbid conditions.

The data presented in this report summarize efforts of a new collaborative group, the Health Economics Research on Cancer Workgroup, to promote health economics research on cancer. The workgroup is composed of scientists from CDC, the National Cancer Institute, Agency for Healthcare Research and Quality, the American Cancer Society, Emory University, and the Livestrong Foundation. The workgroup seeks to address key research gaps identified in IOM reports (4), including the need for national estimates of the burden of cancer, examining the financial impact of cancer on survivors and their families, and patterns of employment. Findings from these studies will provide invaluable information to help improve the quality of the cancer survivorship experience and reduce the burden of cancer in the United States.

With the projected increase in the number of cancer survivors, the economic burden of cancer will also likely increase (3). Therefore, public health decision-makers, professional medical organizations, and other stakeholders might want to focus their efforts on factors that can help to reduce the burden of cancer in the general population, including the recurrence of cancer in cancer survivors. Some of these factors might include primary prevention efforts, such as quitting smoking, being physically active, and maintaining a healthy weight. The economic data presented in this report investigating the economic consequences of surviving cancer highlight the need to develop comprehensive intervention programs to improve the quality of the cancer survivorship experience and decrease the economic burden of cancer survivorship in the United States.

## Figures and Tables

**TABLE 1:** Characteristics of cancer survivors and persons without a cancer history — Medical Expenditure Panel Survey (MEPS), United States, 2008–2011

	2011 MEPS Experiences with, Cancer Survivorship survey		2008–2011 Core MEPS survey			
		
	Cancer survivor (n = 1,202)		Cancer survivor (n = 6,722)		No history of cancer (n = 86,865)	
			
Characteristic	%*	(95% CI)	%	(95% CI)	%	(95% CI)
**Age at interview (yrs)**						
18–39	4.5	(3.3–6.0)	7.1	(6.2–8.2)	41.8	(40.8–42.7)
40–44	3.3	(2.5–4.5)	4.1	(3.4–4.8)	9.3	(9.0–9.7)
45–49	5.3	(4.0–6.9)	5.4	(4.7–6.1)	9.5	(9.1–9.8)
50–54	8.8	(6.9–11.1)	8.2	(7.0–9.6)	9.9	(9.5–10.4)
55–59	10.0	(8.1–12.2)	10.1	(9.1–11.1)	8.3	(8.0–8.6)
60–64	13.6	(11.6–15.6)	12.7	(11.4–14.0)	6.9	(6.5–7.2)
65–69	14.6	(12.3–17.1)	12.4	(11.0–13.9)	4.7	(4.5–5.0)
70–74	12.9	(11.0–15.2)	11.6	(10.5–12.8)	3.3	(3.1–3.5)
75–79	9.3	(7.5–11.5)	10.3	(9.2–11.5)	2.6	(2.4–2.8)
≥80	17.8	(14.8–21.2)	18.2	(16.1–20.6)	3.7	(3.4–4.0)
**Sex**						
Men	42.5	(39.3–45.8)	41.8	(40.0–43.6)	49.0	(48.6–49.4)
Women	57.5	(54.3–60.7)	58.2	(56.4–60.0)	51.0	(50.6–51.4)
**Race/Ethnicity**						
White, non-Hispanic	85.9	(83.5–88.0)	84.8	(83.3–86.2)	66.1	(64.2–67.9)
Black, non-Hispanic	6.6	(5.4–8.0)	6.9	(6.1–7.9)	11.9	(10.7–13.3)
Hispanic	5.1	(3.8–6.7)	5.3	(4.5–6.1)	14.9	(13.4–16.6)
Other, non-Hispanic	2.5	(1.6–3.7)	3.0	(2.2–4.0)	7.1	(6.1–8.2)
**Education**						
Less than high school diploma	13.0	(10.9–15.5)	15.6	(14.2–17.0)	16.9	(16.2–17.7)
High school diploma	29.8	(26.8–32.9)	31.7	(30.0–33.5)	29.5	(28.7–30.3)
Some college or more	57.1	(53.6–60.5)	52.5	(50.4–54.5)	53.2	(52.1–54.3)
**Marital status**						
Married	57.2	(53.4–60.9)	57.7	(54.9–60.4)	52.8	(51.8–53.7)
Not married	42.8	(39.1–46.6)	42.3	(39.6–45.1)	47.2	(46.3–48.2)
**MEPS priority conditions** †						
0	15.8	(13.6–18.4)	16.0	(14.4–17.6)	46.7	(46.0–47.5)
1	18.6	(16.0–21.5)	19.2	(17.7–20.7)	22.6	(22.1–23.0)
2	21.9	(18.7–25.4)	21.3	(19.9–22.9)	14.1	(13.7–14.5)
≥3	43.8	(40.2–47.4)	43.5	(41.7–45.3)	16.6	(16.1–17.1)
**Health status**						
Excellent/Very good	41.4	(38.0–44.9)	39.6	(37.8–41.3)	60.0	(59.1–60.8)
Good	33.7	(30.7–36.9)	32.2	(30.7–33.7)	27.8	(27.2–28.5)
Fair/Poor	24.9	(22.3–27.6)	28.1	(26.4–29.8)	12.2	(11.8–12.6)
**Health insurance or coverage**						
Age <65 yrs, any private	75.2	(70.8–79.1)	74.9	(72.6–77.1)	70.7	(69.5–71.9)
Age <65 yrs, public only	15.8	(12.6–19.5)	14.8	(13.0–16.7)	10.4	(9.8–11.1)
Age <65 yrs, uninsured	9.1	(6.6–12.2)	10.3	(9.0–11.8)	18.9	(17.9–19.9)
Age ≥65 yrs, Medicare and private	62.9	(58.5–67.1)	55.0	(52.1–57.8)	49.9	(47.8–51.8)
Age ≥65 yrs, Medicare and public	5.9	(4.2–8.3)	6.3	(5.2–7.6)	7.9	(7.1–8.9)
Age ≥65 yrs, Medicare only	30.4	(26.4–34.7)	37.8	(35.2–40.5)	40.8	(39.0–42.6)

**Abbreviation:** CI = confidence interval.

* Percentages are weighted using the MEPS Experiences with Cancer Survey weight.

† In addition to cancer, MEPS priority conditions include arthritis, asthma, diabetes, emphysema, coronary heart disease, hypertension, stroke, high cholesterol, angina, and heart attack.

**TABLE 2:** Annual medical expenditures and lost productivity* among cancer survivors and persons without a cancer history — Medical Expenditure Panel Survey (MEPS), United States, 2008–2011†

	Men					Women				
		
	Cancer survivor		No history of cancer			Cancer survivor		No history of cancer		
						
Characteristic	Adjusted mean	(95% CI)	Adjusted mean	(95% CI)	p-value	Adjusted mean	(95% CI)	Adjusted mean	(95% CI)	p-value
**Per capita mean annual medical expenditures**										
**Total expenditures**	**$8,091**	**(7,208–8,974)**	**$3,904**	**(3,741–4,066)**	**<0.001**	**$8,412**	**(7,789–9,036)**	**$5,119**	**(4,955–5,284)**	**<0.001**
**Source of payment**										
Out of pocket	$751	(686–816)	$600	(579–620)	<0.001	$973	(904–1,042)	$833	(807–860)	<0.001
Private health insurance	$3,003	(2,561–3,446)	$1,588	(1,495–1,681)	<0.001	$3,899	(3,384–4,414)	$2,100	(1,993–2,207)	<0.001
Medicare	$1,845	(1,556–2,134)	$1,025	(950–1,100)	<0.001	$1,816	(1,651–1,981)	$1,356	(1,270–1,442)	<0.001
Medicaid	$556	(305–808)	$294	(236–352)	0.005	$720	(566–875)	$484	(442–525)	0.001
Other	$752	(602–903)	$486	(444–527)	<0.001	$671	(552–789)	$402	(370–435)	<0.001
**Service type**										
Ambulatory care	$2,640	(2,344–2,936)	$1,151	(1,102–1,200)	<0.001	$3,187	(2,896–3,478)	$1,689	(1,621–1,758)	<0.001
Inpatient care	$1,722	(1,433–2,011)	$1,289	(1,193–1,385)	0.002	$1,843	(1,615–2,072)	$1,535	(1,441–1,628)	0.003
Prescription medications	$1,343	(1,138–1,549)	$1,077	(899–1,116)	<0.001	$1,650	(1,479–1,820)	$1,186	(1,141–1,231)	<0.001
Other services	$745	(641–848)	$646	(607–685)	0.072	$917	(819–1,015)	$827	(779–874)	0.071
**Per capita mean annual lost productivity**										
**Total productivity loss**	**$3,719**	**(3,123–4,315)**	**$2,260**	**(2,103–2,419)**	**<0.001**	**$4,033**	**(3,519–4,545)**	**$2,703**	**(2,536–2,871)**	**<0.001**
**Source of productivity loss**										
Employment disability	$2,831	(2,433–3,228)	$1,862	(1,739–1,986)	<0.001	$2,961	(2,616–3,305)	$2,109	(1,978–2,241)	<0.001
Missed work days among employed persons	$597	(461–734)	$267	(252–283)	<0.001	$686	(585–787)	$393	(374–413)	<0.001
Lost household productivity	$291	(229–353)	$131	(112–150)	<0.001	$386	(318–453)	$201	(184–217)	<0.001

**Abbreviation:** CI = confidence interval.

* Adjusted to 2011 dollars.

† Estimates are adjusted predicted margins, pooling the MEPS survey weights, based on participants with no missing information for each response. Participants with the responses of “inapplicable,” “refused,” “not ascertained,” or “value assigned, but not collected” were excluded from the analysis. Regression models were adjusted for age (18–39, 40–44, 45–49, 50–54, 55–59, 60–64, 65–69, 70–74, 75–79, and ≥80 years), sex (male or female), marital status (married or not married), race/ethnicity (non-Hispanic white, non-Hispanic black, Hispanic, or non-Hispanic other), MEPS priority conditions (arthritis, asthma, diabetes, emphysema, coronary heart disease, hypertension, stroke, high cholesterol, angina, and heart attack), and education (less than high school diploma, high school diploma, or some college or more).

**TABLE 3:** Indicators of productivity loss in cancer survivors — Medical Expenditure Panel Survey (MEPS) Experiences with Cancer Survivorship Survey, United States, 2011*

	Total†			Men		Women		
				
Indicator	No.	%	(95% CI)	%	(95% CI)	%	(95% CI)	p-value
**Cancer interfered with usual daily activities outside of work**								
Yes	369	31.6	(28.9–34.3)	30.4	(25.6–35.2)	32.5	(28.6–36.4)	0.54
No	759	68.4	(65.6–71.1)	69.6	(64.8–74.4)	67.5	(63.6–71.4)	
**Cancer interfered with ability to perform mental tasks as part of usual daily activities**								
Yes	154	11.6	(9.7–13.6)	10.2	(6.9–13.5)	12.5	(9.6–15.4)	0.35
No	982	88.4	(86.4–90.4)	89.8	(86.5–93.1)	87.5	(84.6–90.4)	
**At any time from when you were first diagnosed with cancer until now, were you employed**								
Yes	676	58.3	(54.9–61.9)	62.4	(57.3–67.6)	55.1	(50.9–59.4)	0.02
No	505	41.6	(8.1–45.1)	37.6	(32.4–42.7)	44.9	(40.6–49.1)	
**Any change in work (extended paid time off, unpaid time off, change in hours, duties, employment status) because of cancer** §								
Yes	285	42.1	(37.9–46.2)	33.7	(26.7–40.7)	48.2	(42.3–54.1)	0.01
No	344	57.9	(53.8–62.1)	66.3	(59.3–73.3)	51.8	(45.9–57.7)	
**Cancer interfered with ability to perform physical tasks required by job** §								
Yes	168	25.1	(20.9–29.2)	26.1	(19.1–33.1)	24.2	(19.7–28.8)	0.08
No	414	65.6	(61.0–70.1)	68.0	(60.6–75.5)	63.3	(58.1–68.5)	
No physical tasks	57	9.4	(6.8–11.9)	5.9	(2.6–9.2)	12.4	(8.4–16.5)	
**Cancer interfered with mental tasks required by job** §								
Yes	103	14.4	(11.4–17.3)	11.5	(6.7–16.3)	16.3	(12.3–20.3)	0.17
No	545	85.6	(82.7–88.6)	88.5	(83.7–93.2)	83.7	(79.7–87.7)	
**Ever felt less productive at work** §								
Yes	169	24.7	(21.0–28.3)	22.2	(15.4–29.0)	26.4	(21.7–31.1)	0.36
No	479	75.3	(71.7–79.0)	77.8	(71.0–84.6)	73.6	(68.9–78.3)	

**Abbreviation:** CI = confidence interval.

* Estimates are adjusted predicted margins, and 95% CIs using the MEPS Experiences with Cancer survey weight, based on participants with no missing information for each response. Participants with the responses of “inapplicable,” “refused,” “not ascertained,” or “value assigned, but not collected” were excluded from the analysis.

† Regression models were adjusted for age (18–39, 40–44, 45–49, 50–54, 55–59, 60–64, 65–69, 70–74, 75–79, and ≥80 years), sex (male or female), marital status (married or not married), race/ethnicity (non-Hispanic white, non-Hispanic black, Hispanic, or non-Hispanic other), MEPS priority conditions (arthritis, asthma, diabetes, emphysema, coronary heart disease, hypertension, stroke, high cholesterol, angina, and heart attack), and education (less than high school diploma, high school diploma, or some college or more).

§ Estimates are based on participants who responded “yes” to the question, “At any time from when you were first diagnosed with cancer until now, were you working for pay at a job or business?”

## Footnotes

* Additional information available at http://www.iom.edu/reports/2013/delivering-high-quality-cancer-care-charting-a-new-course-for-a-system-in-crisis.aspx.
